# Sample storage conditions significantly influence faecal microbiome profiles

**DOI:** 10.1038/srep16350

**Published:** 2015-11-17

**Authors:** Jocelyn M Choo, Lex EX Leong, Geraint B Rogers

**Affiliations:** 1 Infection and Immunity Theme, South Australia Health and Medical Research Institute, North Terrace, Adelaide 5000, SA, Australia; 2 School of Medicine, Flinders University, Bedford Park, Adelaide 5042, SA, Australia

## Abstract

Sequencing-based studies of the human faecal microbiota are increasingly common. Appropriate storage of sample material is essential to avoid the introduction of post-collection bias in microbial community composition. Rapid freezing to −80 °C is commonly considered to be best-practice. However, this is not feasible in many studies, particularly those involving sample collection in participants’ homes. We determined the extent to which a range of stabilisation and storage strategies maintained the composition of faecal microbial community structure relative to freezing to −80 °C. Refrigeration at 4 °C, storage at ambient temperature, and the use of several common preservative buffers (RNAlater, OMNIgene.GUT, Tris-EDTA) were assessed relative to freezing. Following 72 hours of storage, faecal microbial composition was assessed by 16 S rRNA amplicon sequencing. Refrigeration was associated with no significant alteration in faecal microbiota diversity or composition. However, samples stored using other conditions showed substantial divergence compared to −80 °C control samples. Aside from refrigeration, the use of OMNIgene.GUT resulted in the least alteration, while the greatest change was seen in samples stored in Tris-EDTA buffer. The commercially available OMNIgene.GUT kit may provide an important alternative where refrigeration and cold chain transportation is not available.

The importance of the gut microbiome in human health and disease is increasingly clear. Compelling evidence now exists that bacterial colonisation of the gut plays a central role in the development and regulation of the host immunity^1^,^2^, metabolism^3^, and even the gut-brain axis^4^. Disruption of these homeostatic roles through perturbation of the normal microbial balance, a phenomenon referred to as dysbiosis, has been associated with a wide range of pathological conditions; including obesity^5^,^6^, autoimmune diseases^7^, chronic gastrointestinal inflammatory diseases^8^,^9^, type I and II diabetes^10^,^11^ and carcinogenesis^12^,^13^.

Much of the recent insight gained into host-microbiome interactions has been derived through the application of next generation sequencing approaches. In particular, amplicon sequencing targeted to the 16S ribosomal RNA gene provides a relatively inexpensive and rapid characterisation of microbial communities^14^. When applied to faecal material, this approach represents an effective and non-invasive means to assess the gut microbiology, and has been applied across a wide range of research contexts. The ability to detect fine-scale changes in microbiota composition makes 16S gene sequencing useful in assessing changes in longitudinal sample collections, or relatively minor differences between clinically-defined groups. However, the inherent sensitivity of the approach also makes amplicon sequencing particularly susceptible to biases introduced through inappropriate sample handling or storage. For example, faecal microbiota sequencing profiles have been shown to change significantly during ambient temperature storage after 48 hours^15^,^16^. Where performing nucleic acid extraction on fresh samples immediately after collection is impractical, freezing and storage at −80 °C is widely considered to be best practice when preserving microbial composition for sequence-based analysis^17^,^18^,^19^.

Rapid freezing and storage may not always be possible. In many cases, study subjects are requested to collect samples at home. In others, sampling may take place at remote sites where low temperature storage is unavailable. Even where cold temperature storage is available in the form of domestic freezers, the placement of faecal samples in appliances used for food storage is unacceptable to many, and may represent a potential health risk. The cold-chain transfer of samples to processing sites presents additional challenges particularly in the case of large-scale studies, where costs can be prohibitive.

In response, several studies have set out to assess the influence of different sample stabilisation strategies other than ultra-low temperature freezing on the microbial composition of faecal samples. Some effective approaches, such as the immersion of faecal material in ethanol^20^, may preclude many modes of sample transportation, including post and air-freight. Commercial reagents designed for other purposes, such as the RNA preservative, RNAlater, are commonly used as a general preservative, but have been shown to perform relatively poorly in maintaining microbiota composition^20^,^21^. Buffers containing EDTA can inhibit the growth of certain bacteria at concentrations used for sample preservation^22^,^23^, but their impact on sequencing-based microbiota profiling is not well characterised. In addition, OMNIgene.GUT DNA Stabilisation Kit (DNA Genotek), a commercially available approach to temporary preservation of samples at ambient temperature, has recently become available, but its effectiveness relative to other approaches has not been independently assessed. The current absence of data supporting an approach that is appropriate for home faecal sample collection, and non-specialist sample transportation, is therefore a problem for researchers when designing clinical studies with remote collection components.

Our study assessed the extent to which a number of approaches to faecal sample stabilisation could maintain microbiota composition relative to immediate freezing at −80 °C, as determined by 16S rRNA gene sequencing. The approaches evaluated in this study include dry storage at 4 °C (refrigeration) and at ambient temperature, and in suspension in Tris-EDTA (TE) buffer, RNAlater, and OMNIgene.GUT. To minimise the potential of inter-subject variation as a confounding factor^24^, repeat faecal samples obtained from a single subject were analysed. Samples were incubated for 72 hours, a period typical of that associated with the transfer of collected samples to a processing laboratory.

## Results

### Faecal samples alpha diversity

A total of 1,183,513 16S rRNA sequence reads were obtained following quality filtering, equating to 21,917 ± 745 (mean ± SEM) reads per sample. After the removal of chimeras (4.83%), the number of mapped sequence reads ranged from 10,409 to 33,943 (mean ± SEM of 20,612 ± 701) (Supplementary Table 1).

**Figure 1:**
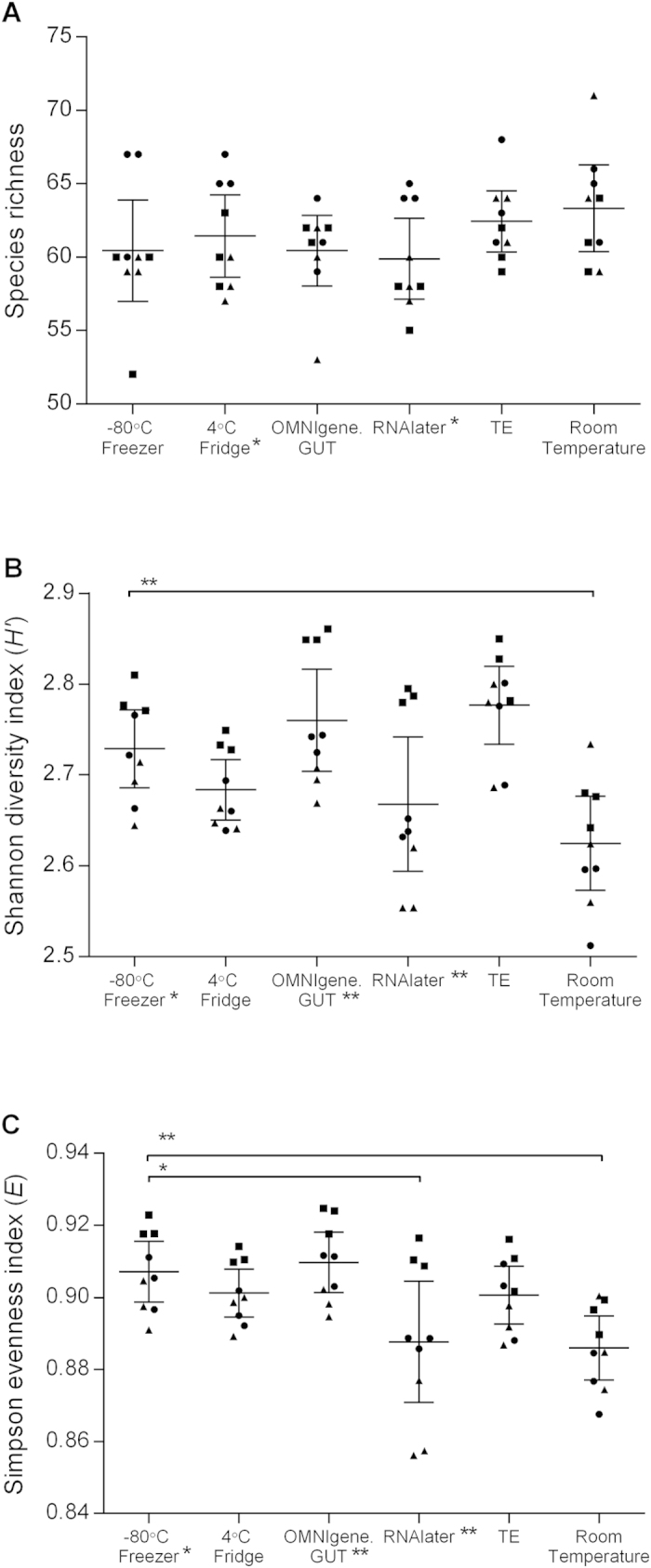
Species diversity following incubation under six different storage conditions . The extent of microbiota structural and composition diversities were measured using (**A**) Taxa S (species richness), (**B**) Shannon-Weiner diversity index, (**C**) Simpson’s evenness index. Each point represents the diversity score for a replicate from collection 1 (●), collection 2 (▲) or collection 3 (■). Error bars represent SEM. Within-group and between-group variations were measured using Kruskal-Wallis one-way ANOVA and Mann-Whitney *U*-test, respectively. Significant variance is indicated by asterisks; single asterisk (*) indicates p ≤ 0.05, double asterisk (**) represents *p* ≤ 0.01.

The effect of storage conditions on faecal microbiota alpha diversity was assessed based on OTU richness (measured based on the absolute number of taxa), diversity (Shannon H’) and evenness (Simpson E). OTU richness did not differ significantly between storage conditions (Fig. 1A). However, samples stored at room temperature had significantly lower Shannon diversity (p = 0.004, Mann-Whitney *U* test) and evenness (p = 0.002) compared to those stored at −80 °C (Fig. 1B,C). Additionally, samples stored in RNAlater had lower evenness compared to −80 °C controls (p = 0.031), whereby a considerable decrease in evenness was observed, particularly for samples in two out of the three collections. Therefore, variation between samples stored using each methodology was also assessed between each of the three collection groups. Significant differences in OTU richness were observed between different collections in RNAlater and at room temperature (p = 0.025, rank-based Kruskal-Wallis test). The diversity and evenness also significantly varied across sample groups that were stored at −80 °C (p = 0.025), in OMNIgene.GUT (p = 0.004) and in RNAlater (p = 0.004).

### Phylum relative abundance

**Figure 2:**
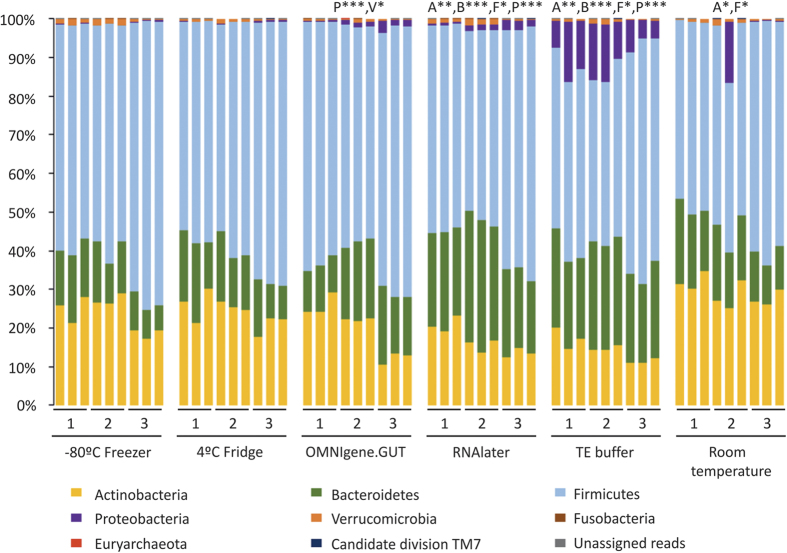
Relative abundance at phylum level for each sample incubated under six different storage conditions. Storage conditions that differed significantly from the control (−80 °C) are indicated with respective phylum abbreviation and asterisks. The respective phyla were abbreviated as follow: Actinobacteria (**A**), Bacteroidetes (**B**), Firmicutes (**F**), Proteobacteria (**P**) and Verrucomicrobia (**V**). Statistical significance was assessed by Mann-Whitney *U*-test and indicated by asterisks; single asterisk (*) represents *p* ≤ 0.05, double asterisk (**) represents *p* ≤ 0.01, and triple asterisk (***) represents p ≤ 0.001.

The dominant phyla across all storage conditions were Firmicutes (median relative abundance, 57.2%; IQR 50.6%, 72.9%), Actinobacteria (21.4%; IQR 15.4%, 26.7%) and Bacteroidetes (18.1%; IQR 13.0%, 22.6%), while a substantial proportion of Proteobacteria was also observed in samples stored in TE solution (9.42%; IQR 5.9%, 1.5%) (Fig. 2). The relative abundance of these four phyla differed significantly when samples were stored in either TE buffer (p < 0.05, Mann Whitney *U* test) or in RNAlater as compared to those at −80 °C (Table 1). A significant increase the relative abundances of Actinobacteria (5.6% ± 2.5%) (p = 0.014) and Firmicutes (10.5% ± 0.9%) (p = 0.019) were also observed in room temperature samples, and Proteobacteria in OMNIgene.GUT samples (1.0% ± 0.4%) (p = 0.0002). No significant variation were detected between the samples stored at 4 °C and −80 °C.

### Microbiota ordination and taxon distribution

**Figure 3:**
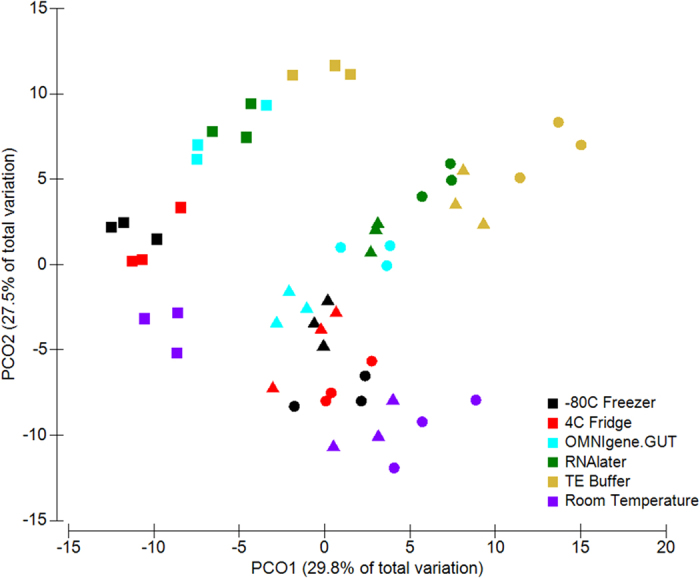
Clustering of samples due to storage conditions by PCoA, based on Bray-Curtis similarity distance. The first two principal coordinates are plotted on the *x*- and *y*-axes, respectively (representing 57.3% of the total variation). Faecal collections sampled at three different time points are represented by circle (●) for collection 1, triangle (▲) for collection 2, and square (■) for collection 3. Storage conditions are indicated by colour.

Principal Coordinate Analysis (PCoA) of Bray-Curtis distances revealed that the samples formed two distinct clusters according to sample collection, with samples in collection 1 and 2 forming one cluster and samples in collection 3 in the other (Fig. 3). Within the sample cluster of collection 1 and 2, samples were separated along the *y*-axis based on the storage conditions, and a similar observation was noted in collection 3. Consistent with these findings, a PERMANOVA (Permutational multivariate analysis of variance) test on the Bray-Curtis dissimilarity matrices indicated that both the collection group (p < 0.0001) and the storage conditions (p < 0.0001) contributed significantly to the differences in microbial composition of the samples. However, the higher pseudo-*F* score of the collection group suggested that differences between the samples has greater effect on the changes observed than differences due to storage conditions (Pseudo-*F*, collection = 34.65; preservation method = 18.24) (PERMANOVA p = 0.0001) (Table 2). These results indicated that the faecal microbiota of the different collection groups were highly heterogeneous; variation that could influence the observed differences between the storage conditions.

Pairwise PERMANOVA tests between each of the assessed storage conditions revealed that only samples stored at 4 °C did not differ significantly in composition compared to those stored at −80 °C (Table 2). Samples from the remaining storage approaches each showed significant divergence to −80 °C controls (PERMANOVA p = 0.0001), with OMNIgene.GUT (*t* = 2.9592) having the least variation among these methods (*t* > 3.3). In addition, microbial composition also differed significantly between samples stored at 4 °C, using OMNIgene.GUT, in RNAlater, in TE buffer, and dry at room temperature (PERMANOVA p < 0.0003) (Supplementary Table 2). Samples stored dry at room temperature (*t* = 3.0168) and using OMNIgene.GUT (*t* = 3.1518) differed least from those stored at 4 °C, while the greatest shifts were observed in samples stored in TE buffer (*t* > 4.4647), consistent with the trend observed when samples were compared to −80 °C storage.

**Figure 4:**
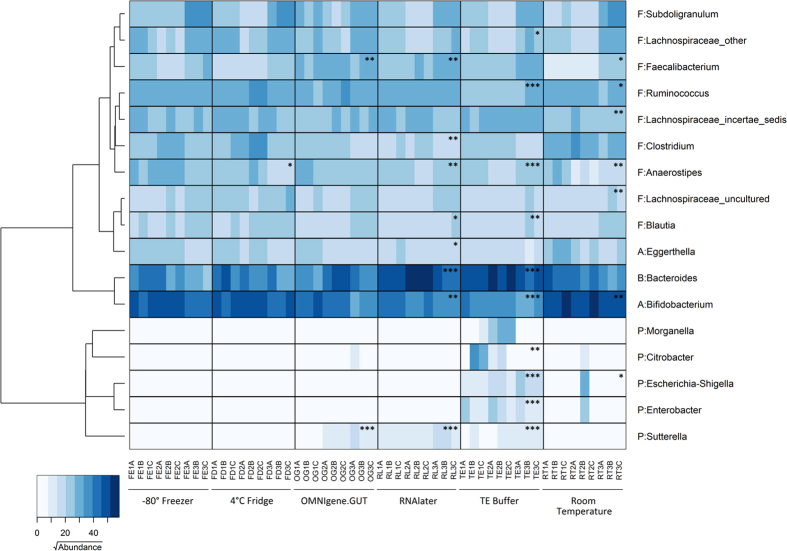
The distribution of the major genera in samples incubated under six different storage conditions. The heatmap shows square root-transformed read counts for the 17 taxa identified as contributing most to variance (86% across all samples) as determined by SIMPER analysis. The dendrogram shows the similarity relationship of genera based on Bray-Curtis distance and Ward’s hierarchical clustering method. Phyla are abbreviated as follows: Actinobacteria (**A**), Bacteroidetes (**B**), Firmicutes (**F**) and Proteobacteria (**P**). Storage conditions that differ significantly from the control (−80 °C) by Mann-Whitney *U*-test are indicated with asterisks; single asterisk (*) represents *p* ≤ 0.05, double asterisk (**) represents *p* ≤ 0.01, and triple asterisk (***) represents *p* ≤ 0.001.

A heat map based on the OTU abundance of the major genera (contributing approximately 86% of the variation as determined by SIMPER analysis) was generated to identify microbial composition that contributed to the variation between samples (Fig. 4). Consistent with the pairwise comparison analysis, changes were observed in the abundance of only a single taxa for the refrigerated samples (*Anaerostipes*, p = 0.024) and two taxa for the OMNIgene.GUT samples (*Sutterella,* p < 0.0001; *Faecalibacterium*, p = 0.001). Several taxa in the samples stored in RNAlater, in TE buffer and at room temperature were also significantly different in abundance to the −80 °C samples. Specifically, a decreased abundance of the genus *Anaerostipes* (p < 0.001) was observed in all three conditions. The abundance of *Bifidobacterium* was lower in the RNAlater (p = 0.004) and TE buffer (p < 0.001) sample groups, but higher in room temperature samples (p = 0.004). The RNAlater and TE buffer samples also had an increased abundance of *Bacteroides* (p < 0.001).

## Discussion

In recent years, there has been increasing interest in understanding the relationship between the gut microbiota and human health. Investigating this relationship has been greatly facilitated by next-generation sequencing^4^,^11^. However, the application of these gene-based approaches can only yield useful insight if the material analysed accurately represents the microbial community at the site sampled. By assessing the impact of a number of commonly used storage conditions on sequencing-based, faecal microbial profiles, we provide an empirical basis for future study design. We used storage at −80 °C as a control based on previous studies that reported no significant variation between fresh samples and those stabilised in this way^18^,^20^.

Substantial interpersonal variation in gut microbiota composition results from differences in factors such as diet^25^ and genetic background^26^. This variation makes discerning the impact of sample storage more difficult when material from multiple individuals is included. For this reason, repeat samples collected from a single subject were used. However, intra-individual variation in sample composition also occurs, and for this reason, we assessed variation between serial samples that were stored at −80 °C. These samples were found to have significant variation in microbial diversity and evenness. PERMANOVA analysis suggested that these variations resulted from differences in sample composition at the different collection timepoints. However, since storage conditions were assessed based on portions of the same starting material, such variation would not be a source of bias.

Freezing has been previously shown to result in a higher ratio of Firmicutes to Bacteroidetes due to changes to the cellular structure of the Gram-positive bacteria when frozen^18^,^19^; a potential confounder given the use of these conditions in our control group. However, all samples in this study were frozen at −80 °C following incubation at the specified conditions. Further, samples were subjected to stringent lysis conditions, including physical disruption, to prevent bias arising from any effects of freeze-thawing.

Our finding that the microbial composition of refrigerated (4 °C) samples did not differ significantly to that of controls stored at −80 °C is in keeping with a previous study performed over a 24 hour period^27^. However, perhaps unsurprisingly, the composition of samples stored at room temperature changed substantially. These changes principally represented increases in the relative abundance of *Bifidobacterium* species, belonging to the phylum Actinobacteria, and decreases in the relative abundance of *Anaerostipes, Ruminococcus, Faecalibacterium* and Lachnospiraceae species, belonging to the phylum Firmicutes. These findings are concordant with a report by Roesch and colleagues^16^ in which they identified a similar decline in Firmicutes bacteria in samples stored at room temperature for 72 hours. The observed shifts in bacterial community structure were also reflected in measures of diversity compared to the −80 °C controls, consistent with the overgrowth of a limited number of species. A number of other prior studies have reported no significant variation in the composition of faecal samples stored at room temperature^15^,^21^. However, it is noteworthy that these studies did not report substantial actinobacterial populations in their subject groups (<5% relative abundance), with greater relative abundances of Bacteroidetes or Firmicutes.

Among the samples stored in stabilisation reagents, the microbiota profiles of those in OMNIgene.GUT diverged least from the −80 °C controls, followed by those in RNAlater, and those in TE buffer (as reflected in the t-statistic of the pair-wise PERMANOVA analysis). The two taxa whose abundance changed significantly in samples stored in OMNIgene.GUT were the *Sutterella* and *Faecalibacterium* genera. This is concerning given the many reports of associations between these genera and a range of gastrointestinal diseases^28^,^29^,^30^,^31^. In samples stored in RNAlater and TE buffer, there was an increase in the Bacteroides population, and a decrease in Firmicutes (*Blautia*), and Actinobacteria (*Bifidobacterium*). In addition, except for the OMNIgene.GUT group, the relative abundance of *Anaerostipes* differed significantly in all storage conditions compared to the −80 °C controls, suggesting that this taxon is particularly sensitive to changes in storage conditions.

Interestingly, we noted an increase in OTU relative abundance for facultative anaerobic Proteobacteria, including *Escherichia-Shigella, Citrobacter and Enterobacter*, in samples stored in TE buffer. The increase in abundance of the facultative anaerobe, Proteobacteria, may be due to exposure of the faecal material to aerobic conditions when suspended in the buffer. Furthermore, these changes (an increase abundance of Proteobacteria and a reduction of Firmicutes and Actinobacteria) resemble those associated with some types of diet-induced gut dysbiosis^32^,^33^,^34^, and as such, we suggest that the use of TE buffer for sample stabilisation should only be considered in combination with low temperature storage to prevent bacterial proliferation.

Samples stored in RNAlater have been shown previously to have decreased DNA purity^21^ and lower DNA extraction yields^20^, potentially affecting downstream analysis. In addition, these studies have reported storage in RNAlater results in significantly lower^21^ or higher^20^ microbial diversity than −80 °C samples (a discrepancy that may be due to differences in analyses being performed on human and non-human primate samples, respectively). Our findings are in keeping with these previous investigations, with room temperature storage in RNAlater resulting in significantly reduced Shannon diversity.

Limitations of our study included the analysis of samples from a single subject, a strategy used to control for inter-subject variation. However, as the impact of storage conditions may vary between individuals, our work could be extended to samples from a larger subject population, with differing gut microbial communities. Further, as new faecal sample stabilisation strategies emerge, it will be important to determine their efficacy relative to the approaches described here.

## Conclusions

Our study highlights several important considerations for studies involving microbiome-wide analysis of faecal samples. Storage conditions have the potential to introduce substantial alterations to microbial community profiling based on 16S rRNA gene sequencing. Where ultra-low temperature storage is unavailable, changes to microbial composition can be minimized by storage and transportation of samples at 4 °C. Changes in microbiota composition in unstabilised samples may explain incongruent findings from previous sequencing-based faecal studies. Significant intra-individual variation occurs in the composition of faecal samples from individual subjects, which is greater than the variation observed between the different storage methods. These findings underline the importance of high frequency, longitudinal faecal collection to understand the temporal dynamics of microbial communities.

## Methods

### Faecal sample collection and processing

Faecal samples were collected from a single healthy volunteer (n = 1) on three different occasions over a three week period with written informed consent. The study protocol was approved by, and carried out in accordance with the guidelines of, the South Australian Health and Medical Research Institute Internal Review Committee. There had been no antibiotic exposure during the collection period, or in the 30 days prior to collection. Each sample was homogenized, and three aliquots stored using each of the six test conditions: freezing at −80 °C, refrigeration at 4 °C, resuspension in a 7× volume of TE buffer (10 mM Tris-HCl pH 8.0, 100 mM EDTA), resuspension in a 7× volume of RNAlater (Ambion, Austin, USA), use of an OMNIgene.GUT stabilisation kit (DNA Genotek, Ontario, Canada), or storage in a dry collection tube. With the exception of samples stored using the OMNIgene.GUT stabilisation kit, all samples were placed in a sterile 2 mL screwcap tube. TE buffer, RNA later and OMNIgene.GUT samples were stored at ambient temperature. All samples were stored in their respective conditions for 72 hours and then frozen at −80 °C prior to DNA extraction, to control for any impact that freezing might have on bacterial community composition.

### DNA extraction and quantification

DNA extraction was performed using MoBio Powerlyzer Powersoil DNA Isolation Kit (MoBio Laboratories, Carlsbad, USA). Samples that were immersed in solution were centrifuged at 13,000 × g for 5 min and the supernatant discarded. Samples were washed with 1 mL of cold 1× PBS (pH7.2) (Life Technologies, Melbourne, Australia) and centrifuged at 13,000 × g for 10 mins. DNA extraction was performed according to the manufacturer’s instructions with the following modifications. Samples were placed into bead tubes with solution C1 and heated at 65 °C for 10 min, prior to two cycles of bead beating at 6.5 m/s for 1 min using a FastPrep-24 bead beater (MP Biomedicals, Santa Ana, USA). Total DNA was eluted in 100 μL of sterile water. DNA concentration was quantified fluorometrically with a Qubit dsDNA HS Assay kit (Life Technologies).

### 16S rRNA gene sequencing

The V4 hypervariable region of the bacterial 16S rRNA gene was amplified from faecal DNA extracts using modified universal bacterial primer pairs 515F (5′-TCGTCGGCAGCGTCAGATGTGTATAAGAGACAGGTGCCAGCMGCCGCGGTAA-3′) and 806R (5′-GTCTCGTGGGCTCGGAGATGTGTATAAGAGACAGGGACTACHVGGGTWTCTAAT-3′), with Illumina adapter overhang sequences as indicated by underline. Amplicons were generated, cleaned, indexed and sequenced according to the Illumina MiSeq 16S Metagenomic Sequencing Library Preparation protocol (http://support.illumina.com/downloads/16s_metagenomic_sequencing_library_preparation.html) with certain modifications. Briefly, an initial PCR reaction contained at least 12.5 ng of DNA, 5 μL of forward primer (1 μM), 5 μL of reverse primer (1 μM) and 12.5 μL of 2× KAPA HiFi Hotstart ReadyMix (KAPA Biosystems, Wilmington, MA, USA) in a total volume of 25 μL. The PCR reaction was performed on a Veriti 96-well Thermal Cycler (Life Technologies) using the following program: 95 °C for 3 min, followed by 25 cycles of 95 °C for 30 sec, 55 °C for 30 sec and 72 °C for 30 sec and a final extension step at 72 °C for 5 min. Samples were multiplexed using a dual-index approach with the Nextera XT Index kit (Illumina Inc., San Diego, CA, USA) according to the manufacturer’s instructions. The final library was paired-end sequenced at 2 × 300 bp using a MiSeq Reagent Kit v3 on the Illumina MiSeq platform. Sequencing was performed at the David R Gunn Genomics Facility, South Australian Health and Medical Research Institute.

### Bioinformatic analysis

The Quantitative Insights Into Microbial Ecology (QIIME, v1.8.0)^35^ software was used to analyse the 16S rRNA sequence generated from paired-end amplicon sequencing using bioinformatics pipeline as previously described^36^. Briefly, barcoded forward and reverse sequencing reads were quality filtered and merged using Paired-End reAd mergeR (PEAR v0.9.6)^37^. Chimeras were detected and filtered from the paired-end reads using USEARCH (v6.1)^38^ against the 97% clustered representative sequences from the Greengenes database (v13.8)^39^. Operational taxonomic units (OTUs) were assigned to the reads using an open reference approach with UCLUST algorithm (v1.2.22q) against the SILVA database release 111 (July 2012)^40^ that was clustered at 97% identity. During the OTU assignment, sequences were pre-clustered at 80% against the reference prior to *de novo* clustering. A minimum subsampling depth of 10,409 reads was selected based on the lowest read depth of samples. QIIME was used to generate an alpha rarefaction curve to confirm that the relationship between read depth and new taxon detection approached an asymptote in all samples at this depth. No samples were eliminated following subsampling.

### Diversity measurements and statistical analyses

PAST (v3.04)^41^ was used to calculate taxa abundance (Taxa_S), Shannon’s diversity index and Simpson’s evenness index for each sample. Beta diversity (Bray-Curtis similarity) was determined using PRIMER (v6, PRIMER-E Ltd, Plymouth, UK). The similarity matrices based on Bray-Curtis distance were calculated using the sample-normalised, square root transformed relative OTU abundance. Principal coordinate analysis (PCoA) was used to visualize clustering of samples based on their similarity matrices. The two-factor permutational multivariate analysis of variance (PERMANOVA)^42^ on the Bray-Curtis matrices was used to test the null hypothesis of no difference amongst a *priori*-defined groups using PERMANOVA + add-on package for PRIMER. The test was computed using unrestricted permutation of raw data with 9,999 random permutations and at a significance level of 0.01.

Variation in microbiota composition at the genus-level was assessed using a two-step approach. First, OTUs that contributed to the overall variation between all six storage conditions were determined using SIMilarity of PERcentages (SIMPER) analysis in PAST. Subsequently, the OTU abundance of the top 17 taxa (accounting for 86% of the dissimilarity between groups) were used to generate a heatmap using gplots package of R statistical software^43^. Hierarchical clustering of the taxa was performed on Bray-Curtis dissimilarity and clustered using Ward’s method. The Kruskal-Wallis one-way ANOVA was used to evaluate the variation among samples in each storage condition. The Mann-Whitney *U* test (two-tailed) was used to compare the variation between the respective storage conditions and −80 °C. Non-parametric statistical analyses were performed using the GraphPad PRISM v6.05 (GraphPad Software Inc., California, USA).

## Additional Information

**How to cite this article**: Choo, J. M. *et al.* Sample storage conditions significantly influence faecal microbiome profiles. *Sci. Rep.* **5**, 16350; doi: 10.1038/srep16350 (2015).

## Supplementary Material

Supplementary Information

## Figures and Tables

**Table 1:** Mean difference in the relative abundance of the phyla Firmicutes Bacteroidetes, Actinobacteria and Proteobacteria in different storage conditions compared to −80°C.

**Phylum**	**Difference in mean relative abundance ± standard error of mean**				
	**−80 **°**C vs 4 **°**C fridge**	**−80 **°**C vs OMNIgene.GUT**	**−80 **°**C vs RNA later**	**−80 **°**C vs TE buffer**	**−80 **°**C vs RT**
Firmicutes	2.4 ± 1.4	3.6 ± 0.8	7.8 ± 1.6	12.7 ± 1.2	10.5 ± 0.9
Bacteroidetes	2.0 ± 0.5	6.9 ± 1.1	13.6 ± 3.2	12.2 ± 2.4	3.6 ± 0.2
Actinobacteria	1.7 ± 0.3	4.1 ± 1.7	7.0 ± 2.4	9.2 ± 1.7	5.6 ± 2.5
Proteobacteria	0.04 ± 0.02	1.0 ± 0.4	1.3 ± 0.4	10.0 ± 2.3	1.8 ± 1.8

**Table 2:** PERMANOVA analysis on Bray-Curtis distance for faecal microbiota structure on the genera level from three different collections and six different storage conditions (permutations = 9999).

**Source**	**df**	**SS**	**MS**	**Pseudo-*****F***	**P(perm)**
Storage	5	3184.9	636.99	18.236	0.0001
Collections	2	2420.9	1210.4	34.653	0.0001
Storage x Collections	10	948.13	94.813	2.7144	0.0001
Residual	36	1257.5	34.93		
Total	53	7811.4			
**Comparison**				***t***	**P(perm)**
−80 °C Freezer vs 4 °C Fridge				1.7381	0.0491
−80 °C Freezer vs OMNIgene.GUT				2.9592	0.001
−80 °C Freezer vs RNAlater				4.6425	0.001
−80 °C Freezer vs TE Buffer				5.6022	0.001
−80 °C Freezer Room Temperature				3.3297	0.001

df = degree of freedom; SS = sum of squares; MS = mean sum of squares; Pseudo-*F* =* F* value by permutation; *t* = *t-*statistic.
