# Supplementary material for: Sample storage conditions significantly influence faecal microbiome profiles
Source: Sci Rep. 2015 Nov 17;5:16350. doi: 10.1038/srep16350 (PMC4648095; doi:10.1038/srep16350)
Supplement: Supplementary Information [file srep16350-s1.doc]

**Sample storage conditions significantly influence faecal microbiome profiles**

Jocelyn M Choo^1†^, Lex EX Leong^1†^, Geraint B Rogers^1,2^*

**Supplementary Table 1: Sequencing depth of each samples from the different storage conditions.**

| **Treatment(s)** | **Collection(s)** | **Replicate(s)** | **Paired-raw reads** | **Mapped reads** |
| --- | --- | --- | --- | --- |
| -80°C Freezer | 1 | A | 19871 | 18678 |
|  |  | B | 15063 | 14162 |
|  |  | C | 19395 | 18219 |
|  | 2 | A | 25254 | 23845 |
|  |  | B | 20424 | 19206 |
|  |  | C | 21147 | 19953 |
|  | 3 | A | 32307 | 30293 |
|  |  | B | 13577 | 12774 |
|  |  | C | 30606 | 28832 |
| 4°C Fridge | 1 | A | 28050 | 26473 |
|  |  | B | 17589 | 16563 |
|  |  | C | 26046 | 24518 |
|  | 2 | A | 23816 | 22542 |
|  |  | B | 20731 | 19418 |
|  |  | C | 23491 | 22123 |
|  | 3 | A | 25886 | 24445 |
|  |  | B | 18800 | 17677 |
|  |  | C | 21125 | 19848 |
| OMNIgene.GUT | 1 | A | 11866 | 11104 |
|  |  | B | 21934 | 20693 |
|  |  | C | 19353 | 18084 |
|  | 2 | A | 11191 | 10409 |
|  |  | B | 16719 | 15640 |
|  |  | C | 15342 | 14318 |
|  | 3 | A | 25513 | 23903 |
|  |  | B | 16230 | 15212 |
|  |  | C | 22366 | 20967 |
| RNAlater | 1 | A | 27394 | 25804 |
|  |  | B | 24777 | 23317 |
|  |  | C | 36048 | 33943 |
|  | 2 | A | 23225 | 22028 |
|  |  | B | 12171 | 11560 |
|  |  | C | 19822 | 18635 |
|  | 3 | A | 14404 | 13497 |
|  |  | B | 20125 | 18852 |
|  |  | C | 21032 | 19678 |
| TE Buffer | 1 | A | 18687 | 17631 |
|  |  | B | 28523 | 26776 |
|  |  | C | 26900 | 25232 |
|  | 2 | A | 24706 | 23311 |
|  |  | B | 27048 | 25479 |
|  |  | C | 22908 | 21554 |
|  | 3 | A | 28244 | 26508 |
|  |  | B | 26625 | 24746 |
|  |  | C | 34145 | 32043 |
| Room Temperature (24°C) | 1 | A | 18497 | 17578 |
|  |  | B | 17036 | 16123 |
|  |  | C | 24021 | 22691 |
|  | 2 | A | 19638 | 18625 |
|  |  | B | 15262 | 14351 |
|  |  | C | 21163 | 19883 |
|  | 3 | A | 21458 | 20274 |
|  |  | B | 21827 | 20395 |
|  |  | C | 24135 | 22645 |
| Mean |  |  | 21917 ± 745 | 20612 ± 701 |

**Supplementary Table 2:** **Pairwise PERMANOVA analysis between all six different storage conditions. Analysis was computed based on the Bray-Curtis dissimilarity distances at the genera level from three different collections.**

| **Groups** | **t** | **P(perm)** | **Unique perms** |
| --- | --- | --- | --- |
| -80°C Freezer vs 4°C Fridge | 1.7381 | 0.0491 | 9936 |
| -80°C Freezer vs OMNIgene.GUT | 2.9592 | 0.0001 | 9958 |
| -80°C Freezer vs RNAlater | 4.6425 | 0.0001 | 9940 |
| -80°C Freezer vs TE Buffer | 5.6022 | 0.0001 | 9935 |
| -80°C Freezer vs Room Temperature | 3.3297 | 0.0001 | 9922 |
| 4°C Fridge vs OMNIgene.GUT | 3.1518 | 0.0001 | 9947 |
| 4°C Fridge vs RNAlater | 4.8419 | 0.0001 | 9959 |
| 4°C Fridge vs TE Buffer | 5.6422 | 0.0001 | 9935 |
| 4°C Fridge vs Room Temperature | 3.0168 | 0.0001 | 9939 |
| OMNIgene.GUT vs RNAlater | 2.7886 | 0.0003 | 9938 |
| OMNIgene.GUT vs TE Buffer | 4.4647 | 0.0001 | 9928 |
| OMNIgene.GUT vs Room Temperature | 4.1946 | 0.0001 | 9938 |
| RNAlater vs TE Buffer | 3.8874 | 0.0001 | 9946 |
| RNAlater vs Room Temperature | 5.13 | 0.0001 | 9932 |
| TE Buffer vs Room Temperature | 5.0669 | 0.0001 | 9951 |
